# The lack of efficacy of tirzepatide in mitigating cisplatin-induced neurotoxicity and cognitive impairment in rats

**DOI:** 10.3389/ftox.2026.1752511

**Published:** 2026-01-16

**Authors:** Hanan Mubarak Almutairi, Ahmad Hamad Alhowail

**Affiliations:** Department of Pharmacology and Toxicology, College of Pharmacy, Qassim University, Buraydah, Saudi Arabia

**Keywords:** antioxidants, cisplatin, cognitive function, oxidative stress, tirzepatide

## Abstract

**Introduction:**

Cisplatin (CIS) is a commonly utilized chemotherapeutic agent, but its use is often accompanied by adverse effects such as neurotoxicity and cognitive impairments, collectively referred to as chemobrain. This condition impacts over 70% of cancer survivors, and currently, there are no established therapeutic interventions. This study aimed to evaluate the efficacy of tirzepatide in mitigating the neuropathy effects induced by cisplatin therapy.

**Methods:**

Forty female Wistar albino rats were divided into four groups of ten: control (untreated), cisplatin (CIS), tirzepatide (TIRZ), and CIS/TIRZ. Treatments were administered intraperitoneally in two injections. The CIS group received cisplatin at a dosage of 5 mg/kg, while tirzepatide was administered at 1.35 mg/kg. In the CIS/TIRZ group, tirzepatide (1.35 mg/kg) was administered prior to cisplatin (5 mg/kg), with a 3-h interval between the two treatments. Post-treatment, behavioral assessments (Y-maze) and oxidative stress biomarkers were evaluated, including enzymatic antioxidants catalase, superoxide dismutase (SOD), and glutathione peroxidase (GPx-1), as well as oxidative damage markers such as reactive oxygen species (ROS) and malondialdehyde (MDA).

**Results:**

Survival rates were 90% in both the TIRZ and CIS groups, and 70% in the CIS/TIRZ group, whereas all rats in the control group survived. All treatment groups experienced a reduction in body weight compared to the control group. Cisplatin administration resulted in impaired learning and memory in the Y-maze test, which was linked to decreased levels of the antioxidants GPx-1 and catalase, with no alteration in SOD levels. Additionally, ROS and MDA levels were slightly elevated in the CIS and TIRZ groups individually. Although tirzepatide did not ameliorate the memory deficits or antioxidant reductions caused by cisplatin, it did lead to a reduction in ROS and MDA levels.

**Discussion:**

CIS therapy accelerates memory deficits in female rats by increasing oxidative stress. However, TRIZ did not alleviate the memory deficits or antioxidant reductions, although it did reduce ROS levels.

## 1 Introduction

A growing number of cancer patients can survive due to chemotherapy, although it is unfortunate that those who undergo treatment often face adverse effects (Tong et al., 2020). Chemotherapy-induced cognitive impairment (commonly known as “chemobrain”) is the term used to describe the slow processing speed, memory impairment, inability to concentrate, and language difficulties that cancer survivors experience (Nelson et al., 2007). Chemotherapy may increase the risk of accelerated aging and possible neurological disease, a growing concern. Treatment with cisplatin usually also leads to cognitive deficits (Chiu et al., 2018). Cisplatin is an effective chemotherapy drug used to treat a variety of cancers, including bladder, ovarian, head and neck, lung, testicular, cervical, esophageal, breast, and brain tumors, and is administered as an injection either alone or with other chemotherapeutic agents. (Li et al., 2019; Dasari and Bernard Tchounwou, 2014). However, because of its severe side effects, which significantly reduce quality of life and affect treatment adherence, cisplatin has limited use in clinical practice (Pini et al., 2016; Florea and Büsselberg, 2011). Cisplatin’s complex and multifaceted anticancer mechanisms include inflammation through activating proinflammatory cytokines, production of reactive oxygen and nitrogen species, activation of multiple signal transduction pathways, including those involving the p53 protein, and cell apoptosis (Faubel et al., 2007; Chtourou et al., 2015). The potential long-term cognitive and behavioral CNS effects are two critical aspects of the nervous system side effects of cisplatin therapy that need to be considered (Troy et al., 2000).

Although the pathophysiology of cisplatin-induced cognitive impairment (CICI) has not been fully elucidated, several pathophysiologic mechanisms, including oxidative stress, inflammation, mitochondrial dysfunction, DNA damage, and apoptosis, have been proposed as possible explanations (Canta et al., 2015; Huang et al., 2021). The principal mechanism of action of cisplatin has been associated with the cross-copper transporters in the cell membrane, which allow cisplatin to enter cells after it first passes through the blood-brain barrier and accumulates in the hippocampus. It is then activated in the cytosol by hydrolysis (Aldossary, 2019; Lomeli et al., 2017; Ongnok et al., 2020). Cisplatin is known to interfere with the mitochondrial complex and disrupt cellular respiration, resulting in increased and unregulated levels of reactive oxygen species (ROS) (Alhowail, 2025). ROS is also formed when equitized cisplatin attacks reduced glutathione (GSH) and metallothionein (MT). Production of ROS induces apoptosis. (Lomeli et al., 2017; Ongnok et al., 2020). A rise in ROS induces nuclear DNA damage at the junction of two adjacent DNA bases, as is mitochondrial DNA damage (Florea and Büsselberg, 2011). Increases nuclear DNA damage, symbolized by joining two adjacent DNA molecules by cross-linking (Lomeli et al., 2017; Ongnok et al., 2020).

Many studies have shown that cisplatin-induced mitochondrial damage in cells leads to oxidative stress and neuronal damage that results in cognitive impairment (CICI) (Canta et al., 2015). Mitochondria also contain a tiny genome for 13 proteins encoded by mitochondrial DNA (mtDNA). Most of these proteins are components of larger complexes required for oxidative phosphorylation in the electron transport chain that produces ATP (Taanman, 1999). In isolated rat neural stem cells and rat hippocampal neurons, cisplatin directly damaged mtDNA and reduced mitochondrial respiratory capacity, causing an elevation of ROS (Lomeli et al., 2017). The increased oxidative stress triggered by mitochondrial dysfunction may have initiated the intrinsic apoptotic process (Ongnok et al., 2020). Oxidative stress occurs when there is an imbalance between free radicals, particularly reactive oxygen species (ROS), and the body’s detoxification or damage repair processes. Free radicals from mitochondrial damage DNA, proteins, and lipids, affecting cellular function and producing malondialdehyde. SOD, an endogenous antioxidant enzyme, converts high ROS concentrations, such as the superoxide anion, into hydrogen peroxide (H_2_O_2_) (Schieber and Chandel, 2014). Glutathione peroxidase (GPx) and catalase turn hydrogen peroxide into water. Therefore, when ROS increased, it is reduced by SOD and GPx and catalase. Consequently, it is reported that alterations in these enzyme levels attenuated their ability to reduce the oxidative stress.

The GLP-1 and GLP-1 receptor pathway have been shown in previous studies to improve cognitive abilities significantly and to have neuroprotective effects by readily crossing the blood-brain barrier (BBB), minimizing oxidative stress, and reducing neuronal cell death in diabetes (El‐Deeb et al., 2020; Cai et al., 2017; Taanman, 1999; Schieber and Chandel, 2014). GLP-1 stimulation of the Akt signaling pathway or downregulation of activating transcription factor 4 were two pathways in which GLP-1 stimulation dramatically reduced stress-induced apoptosis (Cai et al., 2017). Lixisenatide improved neurocognitive abilities, successfully reduced pro-inflammatory mediators, and accelerated the clearance of amyloid plaques in rodent models of Alzheimer’s disease (AD). In addition, GLP-1 agents, a previous study by Koshal et al., reported that liraglutide co-treatment significantly reduced seizure severity, restored behavioral activity, reduced oxidative stress, and restored altered levels of neurotransmitters observed in rodents with comorbidities (Guo et al., 2023). The previous studies mentioned show a significant effect of GLP-1 in alleviating cognitive impairment, proinflammatory mediators, and altering neurochemicals in rodent brains (Reich et al., 2022). Tirzepatide, a GLP-1 agonist, was found to improve spatial learning and memory impairment significantly, inhibit the accumulation, prevent structural damage, increase synaptic protein synthesis, increase dendritic spine formation in the diabetic hippocampus through abnormal changes in signaling molecules related to inflammatory signaling pathways, and restore PI3K/Akt/GSK3 signaling in diabetic rats (Guo et al., 2023). According to studies by Xiying et al., Tirzepatide may be a potential co-therapeutic agent for treating cognitive deficits caused by diabetes in rats (Guo et al., 2023). Their finding revealed that cognitive improvement following TIRZ after using Morris water maze, improves elevation in the glucose levels in the blood, inhibits deposits of amyloid beta in the hippocampus of diabetic rats, and improves reduction in postsynaptic density protein-95 and Synaptotagmin-1 as well as restores PI3K/Akt/GSK3β signaling pathway (Guo et al., 2023).

Despite the frequent occurrence of chemotherapy-induced cognitive impairment (chemobrain) in breast cancer patients and the widespread clinical use of cisplatin, there remains a significant gap in research addressing potential neuroprotective interventions, particularly the role of tirzepatide in attenuating cisplatin-induced memory deficits and oxidative stress. While previous studies have primarily focused on elucidating the molecular and cellular mechanisms through which neuroprotective agents mitigate toxicity in diabetic models (Guo et al., 2023). Investigations exploring tirzepatide as a protective strategy against chemotherapy-related cognitive dysfunction are notably lacking. Accordingly, this study was designed to evaluate the neuroprotective potential of tirzepatide in a cisplatin-induced chemobrain model by assessing cognitive performance through behavioral tests and quantifying oxidative stress markers in rat brain tissues, thereby exploring innovative therapeutic strategies for the prevention and management of chemotherapy-associated cognitive impairment.

## 2 Materials and methods

### 2.1 Animals

Forty female Wistar albino rats, cancer-free (100–200 g) and aged (11–13 weeks), were obtained from the animal house of the College of Pharmacy within Qassim University (Buraydah, Saudi Arabia). They were housed in separate plastic cages as groups (each cage containing four rats) under standard laboratory conditions, maintaining the temperature between 25 °C and humidity between 30% and 70%: a well-ventilated room with a 12-h light–dark cycle (7 a.m.–7 p.m.) and unrestricted access to food and water. Body weight was recorded every other day, and the survival rate of rats was monitored daily. The behavioral test was undertaken during the light phase of the cycle. The ethics was permitted by the deanship for Graduate Studies and Scientific Research at Qassim University (Approval # 23–67-05), and the Institutional Animal Care and Use Committee (IACUC) was followed for all experiments described here. All methods were conducted in accordance with the relevant guidelines and regulations. The study was conducted over a 12-day period.

### 2.2 Chemicals

Cisplatin injection (Celplat™ 50; 50 mg/50 mL) was obtained from Celon Laboratories (Gajularamaram, India). Tirzepatide injection (mounjaro™; 5 mg/0.5 mL) was procured from Eli Lilly Italia (Firenze, Italy).

### 2.3 Drug administration

**FIGURE 1:**
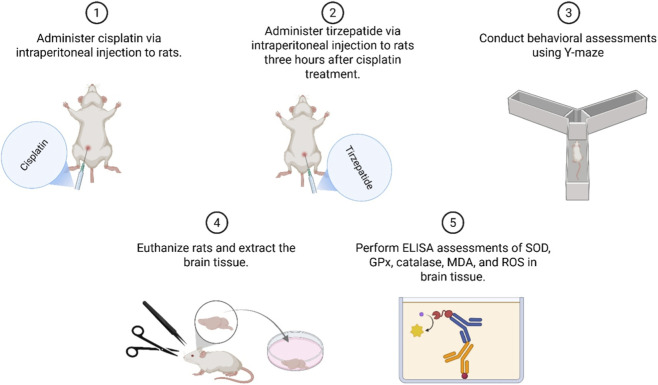
Diagrammatic representation detailing the order and methodology of the research.

Albino Wistar female rats were divided randomly into four groups of 10: TIRZ, CIS, CIS + TIRZ, and control. The sample size (n = 10/group) was selected *a priori* based on group sizes commonly used in comparable behavioral and neuropharmacological studies to ensure adequate power to detect biologically relevant differences while minimizing animal use. Treatment was administered via the intraperitoneal route in two cycles, 3 days apart. According to previous studies (Guo et al., 2023; Okada et al., 2017; Attia et al., 2021), rats were injected with tirzepatide (1.35 mg/kg) in the TIRZ group and cisplatin (5 mg/kg) in the CIS group. The CIS + TIRZ group received a prophylactic dose of tirzepatide (1.35 mg/kg) followed by cisplatin (5 mg/kg), with a 3-h break between the two treatments. The control group received physiologic (0.9%) saline every 3 days for two cycles. At the end of drug administration, the Y-maze test (YMT) was undertaken (Figure 1). The dosing schedule consisted of two intraperitoneal treatment cycles administered 3 days apart. In the CIS + TIRZ group, tirzepatide (1.35 mg/kg, i. p.) was given 3 h before cisplatin (5 mg/kg, i. p.) during each cycle. The Y-maze behavioral assessment was conducted on Day 11 (light phase) following completion of the dosing regimen.

### 2.4 Survival rate and body weight

To ensure animal welfare and research integrity, a rigorous daily monitoring protocol was implemented. This included tracking survival rates, which provided essential data for the study. Cages were sanitized at 48-h intervals, and any deceased animals were immediately removed upon discovery. Systematic monitoring of body weight, a key indicator of general health, was conducted every 48 h. This regular assessment allowed for the detection of minor fluctuations, facilitating the early identification of potential health issues.

### 2.5 YMT

Spatial recognition memory was assessed using a Y-maze task based on the protocol by Kraeuter et al., with modifications to enhance reproducibility and sensitivity in line with current best practices (Kraeuter et al., 2019; Hadzibegovic et al., 2025). All procedures were approved by the Institutional Animal Care and Use Committee (IACUC) of the Deanship of Graduate Studies and Scientific Research at Qassim University under number (23-67-05). Adult male Wistar rats (8 weeks old, 250–300 g) were group-housed in a temperature- and humidity-controlled vivarium (22 °C ± 1 °C, 55% ± 5% humidity) on a 12-h light/dark cycle with *ad libitum* access to food and water. Prior to testing, rats were handled for 15 min daily for 2 days and were acclimated to the testing room for 60 min before the first session. The apparatus was a wooden Y-maze (arms: 50 cm × 10 cm × 18 cm) located in a sound-attenuated room with consistent spatial cues and low-lux lighting (∼25 lux). The maze was cleaned with 70% ethanol between trials.

The behavioral protocol consisted of a 15-min training phase, where the rat could explore two arms while a third (novel) arm was blocked, followed by a 3-h inter-trial interval in the home cage. Subsequently, in a 5-min test phase, the rat was returned to the maze with all three arms accessible. The assignment of the novel arm was counterbalanced across subjects. A ceiling-mounted camera recorded all sessions, and movement was manually tracked using a stopwatch, with the number of entries and time spent in the novel arm calculated. Rats were counted as entering the arm when all four legs were entirely inside the arm. These data were recoded and analyzed using a one-way ANOVA with Tukey’s *post hoc* test; p < 0.05 was considered significant. The experimenter was blinded to experimental conditions during testing and analysis (Alsaud et al., 2023).

### 2.6 ELISAs

Brain tissue samples from all four experimental groups (control, TIRZ, CIS, and TIRZ + CIS) were processed for the quantification of oxidative stress biomarkers and antioxidant enzyme levels. For each group, six biological replicates were randomly selected for analysis.

#### 2.6.1 Sample preparation and protein quantification

Following the completion of behavioral assessments on day 12, rats were euthanized by CO_2_ asphyxiation. Whole-brain tissue samples were harvested immediately post-euthanasia from all experimental groups and stored at −80 °C until further processing. For homogenate preparation, brain samples were homogenized in ice-cold phosphate-buffered saline (PBS, pH 7.4) supplemented with a protease inhibitor cocktail. The homogenates were subsequently centrifuged at 4,000 *g* for 10 min at 4 °C. The resulting supernatants were carefully collected, aliquoted into 200-μL microcentrifuge tubes, and stored at −20 °C for subsequent biochemical analyses. Frozen brain tissues were weighed and homogenized in N-PER lysis buffer (Thermo Scientific, Madison, WI, United States). Homogenization was performed using a Qsonica homogenizer (30 Hz, Newtown, CT, United States). The resulting homogenates were centrifuged at 14,000 x g for 15 min at 4 °C to pellet cellular debris. The supernatant was carefully collected, and the total protein concentration of each sample was determined using a [Specify protein assay, e.g., bicinchoninic acid (BCA) protein assay kit (Thermo Fisher Scientific, Cat. No. 23225)] according to the manufacturer’s protocol. Absorbance was measured at 562 nm using a microplate reader (ELx800; BioTek Instruments, Winooski, VT, USA). Protein concentrations were used to normalize the results of subsequent ELISAs. Aliquots of the protein lysates were stored at −80 °C until further analysis.

#### 2.6.2 Quantification of oxidative stress and antioxidant markers

The levels of the following biomarkers were quantified using commercially available ELISA kits (ABclonal Technology, Wuhan, China):

- Catalase: (Cat. No. RK03551)
- Superoxide Dismutase (SOD): (Cat. No. RK07054)
- Malondialdehyde (MDA): (Cat. No. RK15281)
- Glutathione Peroxidase (GPx): (Cat. No. RK03696)
- Reactive Oxygen Species (ROS): (Cat. No. RK15283)

All assays were performed in 96-well plates according to the manufacturer’s instructions. Samples and standards were added to the appropriate wells and incubated for 2 h at 37 °C, followed by three washes; 100 μL of working biotin-conjugated antibody was then added and incubated for 1 h at 37 °C, the plates were washed three times, 100 μL of working streptavidin–HRP was added and incubated for 1 h at 37 °C, and the plates were washed three times before addition of 100 μL substrate solution and incubation for 20 min at 37 °C in the dark. All samples and standards were assayed in triplicate. Absorbance was measured at 450 nm with background correction at 570 nm using a microplate reader (ELx800; BioTek Instruments), and analyte concentrations were determined by interpolation from a standard curve generated in parallel with the manufacturer-provided standards. Final concentrations were normalized to total protein content and are reported as pg/mg protein.

### 2.7 Statistical analyses

Results were analyzed using Prism 25 (GraphPad; San Diego, CA, United States). Data are presented as mean ± standard error of the mean (SEM). Prior to applying parametric tests, normality was assessed using the Shapiro–Wilk test and homogeneity of variance was evaluated using Levene’s test. Group differences were analyzed using one-way analysis of variance (ANOVA) followed by Tukey’s multiple comparisons test. A *p*-value <0.05 was considered statistically significant.

## 3 Results

### 3.1 Effects of TRIZ and CIS on mortality rate

**FIGURE 2:**
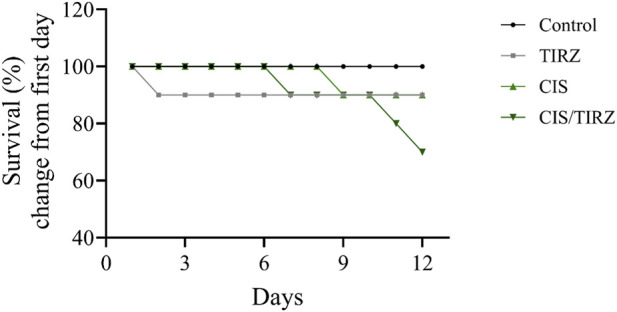
The impact of TIRZ, CIS, and their combined application on the survival rate over the course of the study.

Figure 2 illustrates that all treatment groups experienced a notable reduction in survival when compared to the control group, with a mortality rate of 10% observed in both the CIS and TIRZ groups. In contrast, the combined treatment group CIS/TIRZ exhibited a higher % mortality rate of 30% relative to the control group. The study found no mortalities in the control group.

### 3.2 Effects of TRIZ and CIS on body weight

**FIGURE 3:**
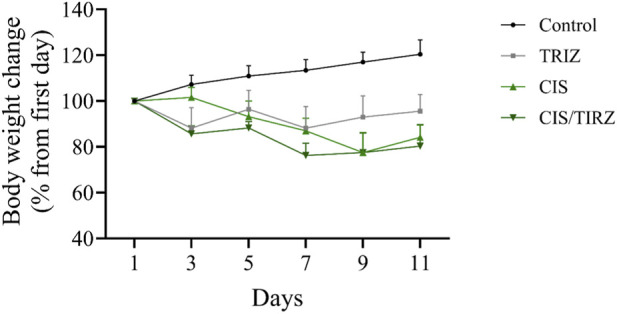
The impact of TIRZ, CIS, and their combined administration on body weight was assessed over the study period. The data are presented as mean values (SEM) and were subjected to analysis using ANOVA, followed by Tukey’s *post hoc* test.

The intervention in the TIRZ, CIS, or CIS/TIRZ groups led to a notable reduction in body weight, approximately 7%, 16%, and 20%, respectively, while the non-treated control group experienced an increase in body weight of around 20% by the study’s conclusion (Figure 3).

### 3.3 CIS combined with TRIZ impairs memory on Y maze task performance

**FIGURE 4:**
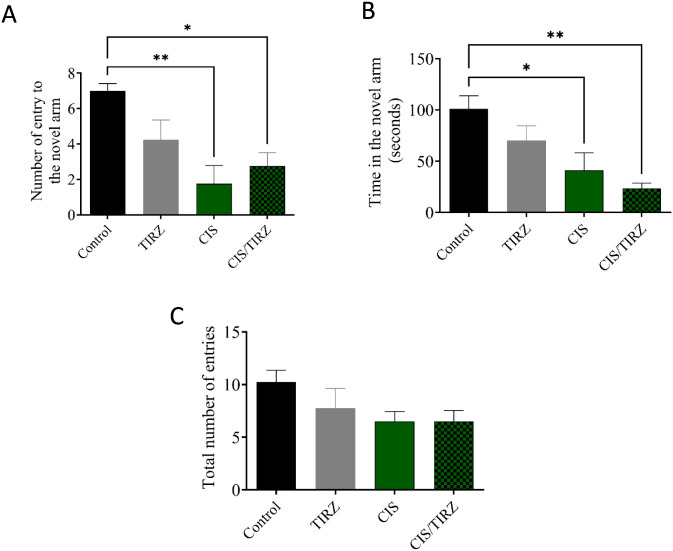
The impact of TIRZ, CIS, and their combination on behavioral tasks was assessed. **(A)** The influence of TIRZ, CIS, and their combination on the frequency of entries into the novel arm was evaluated. **(B)** The effect of TIRZ, CIS, and their combination on the total duration spent in the novel arm was examined. **(C)** The total number of entries into all arms was recorded. Data were presented as mean values (SEM) and analyzed using ANOVA followed by Tukey’s test. *p < 0.05 and **p < 0.01.

The results of the Y maze test, which measures spatial memory, are shown in Figure 6. The number of rats entering the new arm was found to be considerably lower in the CIS and CIS + TRIZ groups compared to the control group (P < 0.01 and P < 0.05, respectively) (Figure 4A). Also, compared to rats in the control group, rats in the CIS group spent considerably less time exploring unfamiliar arms (P < 0.05 for the CIS group and P < 0.01 for the CIS group combined) (Figure 4B). Figure 4C further shows that animals were able to find all arms since the number of entries to each arm remained constant.

### 3.4 The effect of CIS and TRIZ on GPx-1, catalase, and SOD in the brain

**FIGURE 5:**
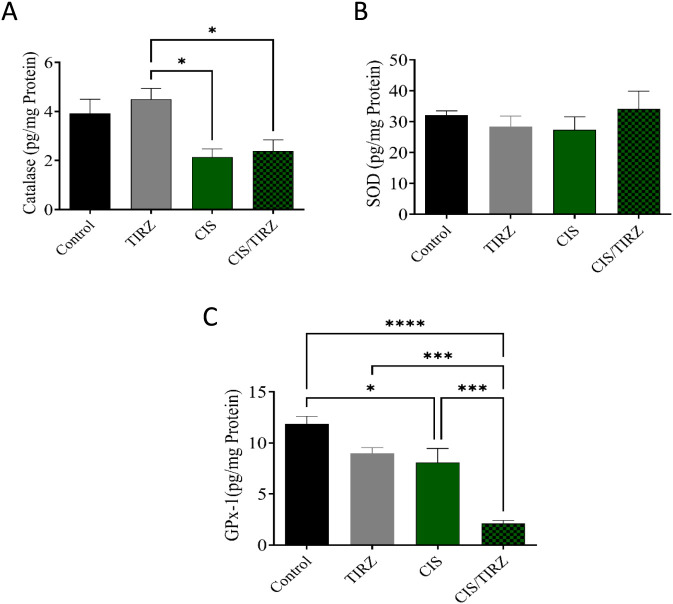
The effects of TIRZ, CIS, and their combination on enzymatic antioxidants in the brain were investigated. Specifically, the concentrations of **(A)** catalase, **(B)** SOD, and **(C)** GPx-1 in the brains of rats were measured. The data are presented as mean values with standard error of the mean (SEM) and were analyzed using ANOVA, followed by Tukey’s *post hoc* test. *p < 0.05, ***p < 0.001, and ****p < 0.0001.

To evaluate TRIZ’s potential in alleviating CIS-induced oxidative stress, the concentrations of endogenous antioxidant mediators (catalase, SOD, and GPx-1) in rat brain tissue were measured using ELISA. Compared to the control group, lower levels of catalase and GPx-1 were detected in the brains of rats in the CIS group (Figures 5A,C). However, combining TRIZ with CIS did not reverse catalase, whereas it had a synergistic effect on GPx-1 compared to the control and CIS alone, and no impact on SOD levels (Figure 5B).

### 3.5 The effect of CIS and TRIZ on ROS and MDA in the brain

**FIGURE 6:**
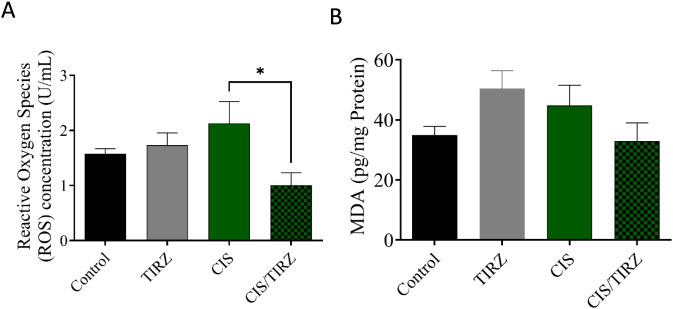
The impact of TIRZ, CIS, and their combined application on oxidative stress biomarkers was investigated. Specifically, the concentrations of **(A)** reactive oxygen species (ROS) and **(B)** malondialdehyde (MDA) were measured. The data were presented as mean values with standard error of the mean (SEM) and subjected to analysis of variance (ANOVA) followed by Tukey’s *post hoc* test.

To evaluate the potential of TRIZ to alleviate CIS-induced oxidative stress, the concentrations of oxidative stress biomarkers (ROS and MDA) in rat brain tissue were measured by ELISA. Compared to the control group, higher levels of ROS and MDA were detected in the brains of rats in the CIS group, and MDA levels also increased in the TRIZ group (Figures 6A,B). However, the combination of TRIZ with CIS reversed these effects and was significant in reducing ROS compared to the CIS group.

## 4 Discussion

The current investigation utilized CIS-treated experimental rats to simulate chemobrain and examine the impact of TRIZ on CIS-induced toxicity, focusing on its influence on oxidative stress and learning and memory behaviors. Multiple lines of evidence have indicated a correlation between chemotherapy treatment and cognitive impairment (Alhowail et al., 2023). While the precise mechanism of this phenomenon remains uncertain, studies suggest that it may be linked to oxidative stress that induces neuronal damage and decreased neurogenesis (Nokia et al., 2012). As a potent chemotherapeutic agent used in chemotherapy regimens, CIS is classified as an alkylating agent, which inhibits DNA synthesis by interfering with DNA replication, leading to cancer cell death and tumor destruction (Dasari and Bernard Tchounwou, 2014). However, it also affects brain neurons, which leads to cognitive impairment (Borchelt et al., 2016). Although the mechanism remains to be fully elucidated, oxidative stress has been implicated in the process by which CIS can induce cognitive impairment (Alhowail, 2025). TRIZ is approved for treatment of obesity and diabetes and has been found to help reduce the cardiotoxicity associated with doxorubicin treatment (Chen et al., 2024). This study examines the effects of CIS on cognitive function in rats and evaluates the potential of TRIZ to mitigate the neuronal toxicity and oxidative stress associated with CIS exposure. Through the evaluation of rats’ performance in behavioral tasks like the Y maze, alongside the measurement of oxidative stress biomarkers ROS, MDA, GPx-1, SOD, and catalase in the brain, a significant disruption in brain function and memory is observed in animals administered CIS, which was not mitigated by concurrent treatment with TRIZ.

Mortality rates and body weight measurements serve as vital indicators of adverse effects, therapeutic efficacy, or drug interactions (Domecq et al., 2015). Throughout the study, all rats in the control group remained alive. In contrast, the survival rate for rats treated with either cisplatin or tirzepatide was 90%. Notably, the combination of CIS and TRIZ resulted in a further reduction in survival, with only 70% of the rats surviving at the end of the study. After day 11 of the study, the body weight of rats in the control group increased by approximately 20% from their initial weight. Conversely, rats in the TRIZ, CIS, and CIS/TIRZ groups experienced significant weight loss, with reductions of approximately 7%, 16%, and 20%, respectively, from their starting weights. These findings collectively suggest that the combination of CIS + TIRZ exerts a more pronounced negative impact than cisplatin alone, despite the inherent toxicity of cisplatin, which affects both survival and body weight. Consequently, our results indicate that TIRZ does not provide any protective advantage when used alongside cisplatin, as it seems to exacerbate rather than alleviate the drug’s toxicity.

Recent studies indicate that chemotherapy involving agents such as cisplatin, doxorubicin, and cyclophosphamide can adversely affect cognitive performance, particularly in tests that rely on hippocampal function (Alhowail, 2025; Borchelt et al., 2016; Alhowail et al., 2021; Ahmed et al., 2023). No approved treatments exist to enhance cognitive function deficits resulting from chemotherapy. This study investigates the mechanism and alterations in cognitive function in rats following the administration of CIS and TRIZ, both individually and in combination, by evaluating their performance in behavioral tasks using a Y-maze. In the Y-maze task, rats administered CIS exhibited a reduction in the frequency of entries into the novel arm and allocated less time exploring compared to the control group. Moreover, the notable decreases in both the number of entries and the duration taken to locate the novel arm by the rats administered the combination of TRIZ and CIS suggest a synergistic effect on memory performance when contrasted with the control group. Overall, the behavioral findings suggest that CIS can lead to cognitive impairment, which is aligned with previous reports (Alhowail, 2025; Park et al., 2024), and that the combination of TRIZ and CIS causes a worsening of this detrimental effect on cognition.

Numerous investigations have demonstrated a link between CIS and neurotoxicity, as well as cognitive decline, through the increase of oxidative stress (Borchelt et al., 2016). A prolonged increase in oxidative stress levels within the brain is associated with the onset of neuronal damage related to apoptosis (Alhowail, 2024). Moreover, CIS can cross the blood-brain barrier and may induce neurotoxic effects by damaging neurons, potentially leading to oxidative stress due to increased levels of ROS and decreased natural antioxidant defenses (Elmorsy et al., 2024). According to prior research, the study’s findings indicated a reduction in dendritic spine density in the brains of mice that were administered CIS (Andres et al., 2014). The findings from the current study indicated a notable reduction in GPx-1 and catalase levels, alongside a minor elevation in ROS levels following CIS treatment when compared to the control group. Furthermore, the reduction of antioxidant proteins in rats subjected to CIS treatment has been associated with cognitive decline. Moreover, studies conducted on experimental rodents and humans have demonstrated that TRIZ can effectively prevent cardioprotective effects by activating the PI3K/Akt signaling pathway, which improves cardiac function. In contrast to these findings, the combination of TRIZ and CIS in the present study resulted in a synergistic effect that adversely affected cognitive function and reduced antioxidants in the brain. However, TRIZ alone did not succeed in restoring the levels of the antioxidants GPx-1 and catalase; surprisingly, TIRZ was observed to mitigate the levels of reactive oxygen species (ROS) induced by CIS. This observation underscores the need for further investigation into the intricate relationship between the reduction of reactive oxygen species (ROS) and the subsequent decline in antioxidant levels.

This investigation reveals a range of strengths and weaknesses. This study represents the inaugural investigation into the impact of TRIZ on cognitive function induced by CIS. Furthermore, it explored the possible connection between TRIZ and CIS therapies and oxidative stress. All subjects in this investigation were carefully matched for age and strain to reduce potential bias, and each trial was executed simultaneously. Furthermore, all subjects were devoid of cancer to facilitate a focused assessment of the distinct impacts of CIS treatment, minimizing potential confounding factors associated with malignancy. The intervention consisted of delivering several doses, akin to the methodology applied in oncology cases. Moreover, the histopathology examination would yield more compelling evidence of oxidative stress within the brain tissues; however, these investigations faced challenges due to insufficient experience in conducting histopathology examinations and the constraints of the laboratory in executing this technique.

## 5 Conclusion

This study substantiates that CIS leads to cognitive impairment, as demonstrated by Y maze behavioral assessments, and links this effect to elevated ROS levels and reduced enzymatic antioxidant biomarkers, namely, GPx-1 and catalase. This finding suggests a possible mechanism for chemobrain. Additionally, while the combination of TIRZ with CIS successfully reversed ROS levels, it did not ameliorate the reduction in enzymatic antioxidants, indicating only a partial mitigation of oxidative stress. It is crucial to emphasize that this study does not undermine the potential of TRIZ, which continues to show promise for therapeutic applications, although its impact on antioxidant enhancement remains limited. Ultimately, this research provides valuable insights into the effectiveness of TIRZ on CIS-induced neuronal dysfunction and serves as a resource for researchers exploring drug interactions in the context of CIS-induced chemobrain.

## Data Availability

The original contributions presented in the study are included in the article/supplementary material, further inquiries can be directed to the corresponding author.
